# Antifouling Behavior of Copper-Modified Titania Nanotube Surfaces

**DOI:** 10.3390/jfb14080413

**Published:** 2023-08-04

**Authors:** Aniruddha Vijay Savargaonkar, Amit H. Munshi, Paulo Soares, Ketul C. Popat

**Affiliations:** 1 Department of Mechanical Engineering, Colorado State University, Fort Collins, CO 80523, USA; aniruddha.savargaonkar@colostate.edu (A.V.S.); amit.munshi@colostate.edu (A.H.M.); 2 Department of Mechanical Engineering, Pontifícia Universidade Católica do Paraná, Curitiba 80215-901, PR, Brazil; pa.soares@pucpr.br; 3 School of Biomedical Engineering, Colorado State University, Fort Collins, CO 80523, USA

**Keywords:** titania nanotube surfaces, antifouling behavior, biofilm formation

## Abstract

Titanium and its alloys are commonly used to fabricate orthopedic implants due to their excellent mechanical properties, corrosion resistance, and biocompatibility. In recent years, orthopedic implant surgeries have considerably increased. This has also resulted in an increase in infection-associated revision surgeries for these implants. To combat this, various approaches are being investigated in the literature. One of the approaches is modifying the surface topography of implants and creating surfaces that are not only antifouling but also encourage osteointegration. Titania nanotube surfaces have demonstrated a moderate decrease in bacterial adhesion while encouraging mesenchymal stem cell adhesion, proliferation, and differentiation, and hence were used in this study. In this work, titania nanotube surfaces were fabricated using a simple anodization technique. These surfaces were further modified with copper using a physical vapor deposition technique, since copper is known to be potent against bacteria once in contact. In this study, scanning electron microscopy was used to evaluate surface topography; energy-dispersive X-ray spectroscopy and X-ray photoelectron spectroscopy were used to evaluate surface chemistry; contact angle goniometry was used to evaluate surface wettability; and X-ray diffraction was used to evaluate surface crystallinity. Antifouling behavior against a gram-positive and a gram-negative bacterium was also investigated. The results indicate that copper-modified titania nanotube surfaces display enhanced antifouling behavior when compared to other surfaces, and this may be a potential way to prevent infection in orthopedic implants.

## 1. Introduction

Orthopedic implants are one of the most essential implants for the body as they affect the quality of life of patients with bone and cartilage diseases. According to the Center for Disease Control (CDC), the rate of total knee replacement increased by 86% for men and 99% for women aged 45 and over between the years 2000 and 2015, whereas the rate of total hip replacement increased from 12% to 17% for patients aged 45 and over between the years 2000 and 2010 [1]. Implant failure can lead to major complications like aseptic loosening and infection in patients. About 2% to 5% [2] of implants in orthopedic applications are at risk of infection. With the rise in the number of people having these implants, the number of infections has also risen. Infections occur when bacteria attach to the proteins on the implant surface instead of the mammalian cells attaching to these surfaces. Following adhesion, bacteria secrete an extracellular matrix that leads to biofilm formation. Biofilm is a natural exopolymer that is extremely difficult to eradicate despite aggressive antibiotic treatment therapies. *Staphylococcus aureus* is a gram-positive strain of bacteria that is responsible for two-thirds of infections and is the major cause of osteomyelitis, as well as septic arthritis and prosthetic joint infections [3]. It is also the most notorious antibiotic-resistant bacteria [4]. *Pseudomonas aeruginosa*, a gram-negative strain of bacteria, is responsible for 8% of total implant infections [5]. Apart from preventing infections, another important factor is the ability of the implant to successfully osseointegrate with the bone tissue. Osseointegration is defined as the formation of new bone on the implant surface that results in a structural connection and is the prerequisite for the long-term success of orthopedic implants [6].

To prevent infection after orthopedic implant surgery, the patients are clinically prescribed lengthy antibiotic therapy (typically 2 weeks of IV antibiotic treatment followed by oral antibiotic treatment for 3 months in the case of hip prostheses and 6 months for knee protheses [7]). The patients are prescribed antibiotic drugs such as cephalosporins, aminoglycoside, glycopeptides, and quinolones [4]. However, misuse or over-use of antibiotic drugs has led to the development of bacterial resistance towards them, making them non-functional. Antibiotic drugs cannot penetrate the biofilm formed by bacteria on the implant surface, and this leads to removal of the implant for revision surgery, which is even more painful for patients than the initial implant surgery. Around 17.5% of total hip arthroplasties and 8.2% of total knee arthroplasties are revision surgeries [2]. The long-term clinical success of these implants depends on preventing infection on them [8]. Several approaches have been researched with the goal of minimizing bacteria adhesion and growth, and the subsequent prevention of biofilm on implant surfaces. Various modifications for implants have been proposed, such as modifying the surface chemistry and/or modifying the surface topography [9,10,11,12]. Several micro- and nanoscale topographies have been investigated, such as nanowires, nanotubes, nanoflowers, nanorods, and nanoribbons [13,14,15,16,17]. Implant surfaces modified with nanoparticle layers have shown a higher growth of mesenchymal stem cells, as well as a decrease in bacterial growth by disrupting the bacterial membrane [8,18,19].

Titanium and its alloys are commonly used to fabricate orthopedic implants due to their excellent mechanical properties, good corrosion resistance, and biocompatibility [8]. The titania nanotube surface, fabricated using simple anodization process, is one of the nanoscale surface modifications that has demonstrated lower adhesion and growth of bacteria while encouraging mesenchymal stem cell adhesion, proliferation, and differentiation [20,21]. Furthermore, titania nanotube surfaces with different nanotube diameters that have been annealed at different temperatures have demonstrated a reduced number of live and dead bacteria adhered on the surfaces [8]. Titania nanotube surfaces are also hydrophilic and have demonstrated enhanced osseointegration [22]. Research shows that titania nanotube surfaces with an 80 nm diameter have an optimum proliferation and differentiation of osteoblasts [23]. Recently, there has been significant interest in modifying the surface chemistry of implants with metal ions such as copper, silver, zinc, and gold, as well as other biomimetic ceramics such as hydroxyapatite, bio-glass, and calcium phosphates [24,25]. Metal organic frameworks are another surface chemistry modification that have displayed enhanced osteogenic as well as antibacterial activity when used in combination with porous titanium [26]. Despite copper having good antibacterial properties, not many studies have demonstrated titania nanotube surfaces modified with it [24,27]. Copper is an important micronutrient required for normal innate and adaptive immune system functioning. When bacteria encounter surfaces that are modified with copper, a phenomenon known as contact killing occurs. The bacterial membrane is damaged, leading to DNA degradation, resulting in the death of bacteria [28,29]. Contact killing is also very rapid and prevents the bacteria from proliferating quickly on the copper-modified surfaces. However, if the release of copper ions is high, it may be toxic to the cells within the body [29].

Postoperative infections are, hence, a significant cause of revision surgeries in orthopedic implants. The current approach to resolve this includes prescribing the patients with antibacterial drugs like quinolones. However, there are complications and side effects, such as skin allergy, gastrointestinal side effects, and making the bacteria themselves drug-resistant [30]. An alternate approach to preventing infection that is not dependent on antibacterial drugs could be to modify the implant surfaces with agents that have antibacterial properties. Recently, research has shown copper deposited on surfaces has antibacterial properties [31,32]. 

In this study, copper-modified titania nanotube surfaces were fabricated using a simple anodization process followed by the physical vapor deposition of copper and subsequent annealing of the surfaces. Following the fabrication, the surfaces were characterized through scanning electron microscopy (SEM) to evaluate surface morphology, energy-dispersive X-ray spectroscopy (EDS), and X-ray photoelectron spectroscopy (XPS) for surface chemistry, contact angle for surface wettability, and X-ray diffraction for surface crystallinity. The surfaces were evaluated for their antifouling behavior against gram-positive (*Staphylococcus aureus*) and gram-negative (*Pseudomonas aeruginosa*) bacteria. The surfaces were characterized using fluorescence microscopy and SEM to evaluate the bacteria’s adhesion, growth, and morphology. The results indicate that copper-modified titania nanotube surfaces displayed enhanced antifouling behavior when compared to other surfaces, and this may be a potential way to prevent biofilm formation on implant surfaces.

## 2. Materials and Methods

### 2.1. Fabrication of Copper-Modified Titania Nanotube Surfaces

Titania nanotube surfaces (NT) were fabricated on commercially pure medical grade titanium (Ti) foils using an anodization and annealing process [33,34,35]. The Ti foils were cut into a size of 25 mm × 25 mm × 0.5 mm and mechanically polished using silicon carbide sheets of different grit sizes (200, 400, 600, 800, and 1000). The polished Ti surfaces were cleaned by sonicating them in acetone, followed by rinsing in soap solution, isopropyl alcohol, and de-ionized (DI) water. The cleaned Ti surfaces were anodized at 55 V in an electrolyte solution containing 95% diethylene glycol (DEG), 3% DI water, and 2% hydrofluoric acid for 22 h. A two-electrode setup was used with Ti surfaces as anode and platinum foil as cathode. After anodization, the surfaces were rinsed in DI water and isopropyl alcohol, followed by annealing at 530 °C for 3 h in an ambient oxygen environment. The temperature increment rate was 15 °C/min at the beginning of the annealing process. 

### 2.2. Copper Deposition on Titania Nanotube Surfaces

The copper modification of surfaces was carried out using a novel one-step physical vapor deposition (PVD) method. Copper chloride (CuCl_2_) was used as a source for copper deposition on the surfaces. Prior to deposition, the vacuum chamber was first pumped down to about 5 × 10^−5^ Torr base vacuum and then argon was used as the carrier gas to maintain 40–60 mTorr vacuum. Ti and NT surfaces were washed with deionized (DI) water and dried with argon gas. The Ti or NT surfaces were placed in a quartz adapter plate and then introduced into a vacuum load-lock where the adapter plate was secured on a sample manipulator. The load-lock was evacuated using a mechanical pump to match the pressure of the main PVD chamber of <40 mTorr. The load-lock isolation gate valve was then opened and using a magnetic transfer mechanism, the sample manipulator was introduced into the main PVD chamber. Preheating was carried out at 330 °C for 2 min in a vacuum chamber at ~65 mTorr pressure. After preheating, the Cu deposition was carried out at the crucible temperature of 190 °C and the surfaces were then annealed at 200 °C, allowing diffusion of Cu into the surfaces. All three process steps included an additional heater located on top of the crucible that enabled reliable control of surface temperature during processing, thus maintaining a controlled and repeatable temperature gradient. The heater temperature was maintained at 330 °C, 170 °C, and 200 °C for preheating, CuCl_2_ deposition, and annealing, respectively. 

### 2.3. Characterization of Copper-Modified Titania Nanotube Surfaces

Morphological and topographical features of the different surfaces were characterized using a JEOL 6500 field emission scanning electron microscope (SEM). Prior to collecting the SEM images, surfaces were coated with 10 nm gold (Au) using a sputter coater to enhance the conductivity. The SEM parameters were optimized and chosen as follows: accelerating voltage of 15 kV, working distance range of 7–10 mm, and vacuum pressure below 3 × 10^−4^ Pa. The images were collected at 5000× magnification. 

Surfaces prepared for SEM were also examined for surface elemental composition and elemental distribution using an energy-dispersive X-ray spectroscopy (EDS) probe (Thermo Electron, Noran system) attached to the SEM. EDS was used to detect the presence of copper on the surfaces. Surfaces were analyzed for 5 min at 5–15 kV and a magnification of 5000× to provide a complete profile of different elements present. 

The surface wettability was characterized by taking static contact angle using a Ramé-Hart 260F4 goniometer. A 10 μL drop of DI water was placed on the surface and the contact angle was measured after 3 secs using the DROPimage software.

The surface chemistry was characterized using PHI-5600 X-ray photoelectron spectroscopy (XPS) probe equipped with Al Kα X-ray source. Survey spectra were collected at a pass energy of 187 eV and a 0.05 eV step for 16 cycles. The data were analyzed, and percentage of elements present on the surface was calculated using CASA XPS software. The high-resolution spectra scans were collected for copper (Cu2p3) on different surfaces. The scan was carried out using a pass energy of 23 eV and a 0.05 eV step for 30 cycles.

The surface crystallinity was characterized using thin-film X-ray diffraction (TF–XRD, Shimadzu, XRD-7000). Spectra were collected using CuKα radiation (λ = 1.5406 Å) with continuous scanning at a scanning speed of 1°/min, over the 2θ range of 20° to 80° at an incidence angle of 5°.

### 2.4. Cytotoxicity of Different Surfaces

The cytotoxicity of different surfaces was evaluated using a lactate dehydrogenase (LDH) assay (CyQUANT^TM^ LDH Cytotoxicity Assay Kit). The cytotoxicity was evaluated using adipose-derived stem cells (ADSCs). The cells were grown in culture flasks containing ⍺-MEM media containing 10% Fetal Bovine Serum and 1% Penicillin-Streptomycin solution. After achieving 80% confluency, 20,000 cell/mL were transferred into each well of a 48-well plate containing different surfaces. Prior to cell culture, all the surfaces were sterilized using ultraviolet (UV) light for 1 h and washed in phosphate-buffered saline (PBS) twice. The cells were grown for 24 h at 37 °C and 5% CO_2_. Cells exposed to lysis buffer were used as positive control and cells grown in the well plate were used as negative control. A 50 μL solution from each well plate was placed into a 96-well plate and the protocol provided by the manufacturer was followed to determine the cytotoxicity of different surfaces. 

### 2.5. Bacterial Culture on Copper-Modified Titania Nanotube Surfaces

Two different strains of bacteria, *Staphylococcus aureus* (gram-positive) and *Pseudomonas aeruginosa* (gram-negative) were used to investigate the antifouling behavior of different surfaces. The bacteria were grown in 8 mL tryptic soy broth (TSB) and incubated for 12 h at 37 °C. The bacterial solution was diluted until an optical density reading of 0.52 (i.e., a concentration of 10^9^ Colony Forming Unit (CFU)/mL of TSB solution) was observed at a wavelength of 562 nm. The solution was further diluted to a concentration of 10^6^ CFU/mL of TSB to evaluate adhesion and growth of bacteria on the surfaces. Prior to culturing bacteria, the surfaces were first cleaned with DI water and sterilized under UV light for 60 min. Then the surfaces were transferred to a 24-well plate, with 500 μL bacteria solution of 10^6^ CFU/mL concentration in each well and incubated at 37 °C for 6 h and 24 h.

Fluorescence microscopy was used to characterize bacterial adhesion on different surfaces. After the incubation period, the bacterial solution was removed, and the surfaces were rinsed three times with PBS to remove any unadhered bacteria from the surfaces. Then, surfaces were immersed in a stain solution (3 μL/mL of propidium iodide and Syto 9 stain 1:1 in PBS) and incubated for 15 min in a dark environment. After the incubation, the stain was removed, and samples were rinsed three times with PBS. The bacteria on the surfaces were fixed by placing them in 3.7% formaldehyde solution for 15 min and then rinsing three times with PBS. Finally, a fluorescence microscope (Zeiss Axiovision) was used for imaging the surfaces at 20× magnification.

Bacterial morphology and biofilm formation on different surfaces were characterized using SEM images. After the incubation period, the bacterial solution was removed, and the surfaces were rinsed with PBS. Bacteria on the surfaces were fixed by immersing the surfaces in a fixative solution containing 3% glutaraldehyde, 0.1 M sucrose and 0.1 M sodium cacodylate in DI water for 45 min. Once the bacteria were fixed, they were allowed to sit in a buffer solution (fixative solution without glutaraldehyde) for 10 min. The surfaces were immersed subsequently in 35%, 50%, 70%, and 100% ethanol solutions for 10 min each. Finally, the surfaces were stored inside a desiccator before taking SEM images. Prior to imaging, the surfaces were coated with 10 nm gold (Au) to increase the conductivity. SEM images were captured at 2500× and 10,000× magnification. The following imaging parameters were chosen: accelerating voltage of 15 kV, working distance of 10 mm, and vacuum pressure below 3 × 10^−4^ Pa. 

### 2.6. Statistical Analysis

The surface characterization (SEM, EDS, XPS, and contact angle, except XRD) was performed on least three different surfaces (nmin = 3) for each group. Bacterial adhesion, morphology, and inhibition were performed three times with at least three different surfaces in each group (nmin = 9). The quantitative results were analyzed by two-way analysis of variance (ANOVA) and Tukey’s honestly significant difference (HSD) test using the JMP software with significant results considered when *p* < 0.05.

## 3. Results and Discussion

Titania nanotubes surfaces were fabricated using a simple one step anodization process. During the anodization process, titanium oxidizes to TiO_2_ due to the interaction of metal with O^2−^ or OH^−^ ions, forming an oxide layer. Titanium dissolution also occurs due to the presence of acid in the electrolyte. For the fabrication of titania nanotube surfaces, the presence of hydrofluoric acid in electrolyte plays a vital role. Competition between the oxidation and chemical dissolution leads to the formation of nanotubes from the surface as the oxide growth moves upwards [36]. The nanotube properties depend on three parameters: voltage, electrolyte concentration, and time of anodization. In this study, a voltage of 55 V was used for 22 h, which resulted in nanotubes with an approximate diameter of 80 nm. These nanotubes have demonstrated enhanced cell adhesion in previous studies [23,35]. 

**Figure 1:**
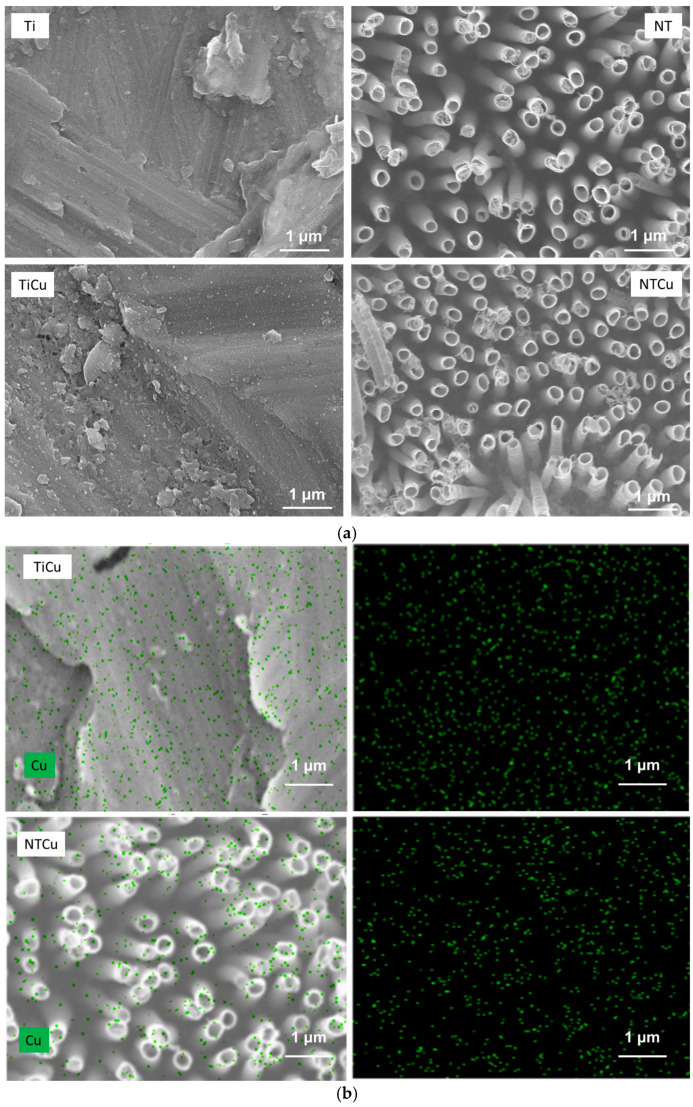
(**a**) Representative SEM images of different surfaces. (**b**) Representative SEM images and corresponding EDS mapping of copper for different surfaces.

The surface topography plays a very important role in interaction with proteins and cells, and hence was characterized by SEM (Figure 1a). The SEM images were also taken for unmodified surfaces to compare them with modified surfaces. As expected, the Ti surfaces do not have any distinct features and look rough due to the processing of the material. However, after anodization, the NT surfaces were very uniform with nanotubes evenly distributed and perpendicular to the surface. Some of the nanotubes were clustered due to surface charge, but overall, the surface was very uniform (Figure 1a). Once surface topography was characterized, they were further modified with copper using a PVD method. Copper from CuCl_2_ sublimates on heating and is deposited on the surface. Further annealing allowed the diffusion of Cu into the surface. The results indicate that TiCu surfaces were still very rough; however, they had slight changes in the surface topography due to copper modification, but not significantly different to Ti surfaces (Figure 1a). The NTCu surfaces were also very similar to NT surfaces, indicating that PVD modification did not significantly alter the surface nanotopography (Figure 1a). The surface topography plays an important role in its effectiveness for cell adhesion, proliferation, and differentiation. Thus, maintaining the nanotopography after the copper modification of NT surfaces is critical for the long-term success of implants.

All the surfaces were further characterized with EDS to determine the distribution of elemental Cu on them. EDS is a quick, non-destructive method that provides an elemental surface map. The results indicate that copper was uniformly present on both TiCu and NTCu surfaces regardless of their topography (Figure 1b).

**Figure 2:**
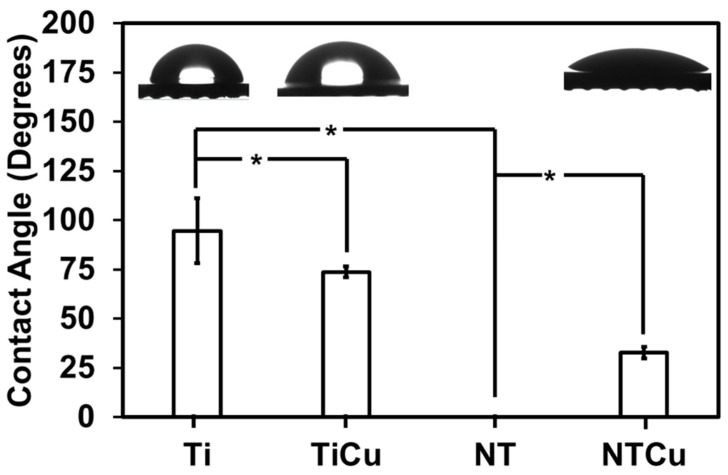
Contact angles for different surfaces with the representative images of water droplets on the surface. Statistical significance (*p*-value) was represented as * *p* < 0.05.

The surface wettability plays an important role in the interaction of proteins and cells with the biomaterial surface. Hydrophilic surfaces tend to have enhanced interaction between the material surface and biological entities, whereas hydrophobic surfaces tend to avoid that interaction. Surfaces with high wettability (smaller contact angles) demonstrate higher mammalian cell attachment and spreading, whereas surfaces with low wettability (larger contact angle) repel cell attachment and spreading [37]. Hence, it is important to evaluate the surface wettability of the surfaces. In this study, contact angle goniometry was used to measure the surface wettability and water contact angles were measured (Figure 2). The results indicated that the Ti surfaces were slightly hydrophobic with a contact angle of 94.5° + 16.52°. After modification with copper, the contact angle of TiCu surfaces decreased to 73.6° + 2.74°, indicating the surfaces were hydrophilic. After anodization, the NT surfaces had a contact angle <10°, indicating the surfaces were superhydrophilic. The superhydrophilicity was due to the nanotube surface topography, which results in a highly porous, high-surface-area surface that leads to complete spreading of the water drop once in contact with the surface. After modification with copper, the contact angle of NTCu surfaces increased to 32.7° + 2.96°, indicating the surfaces were hydrophilic. 

**Figure 3:**
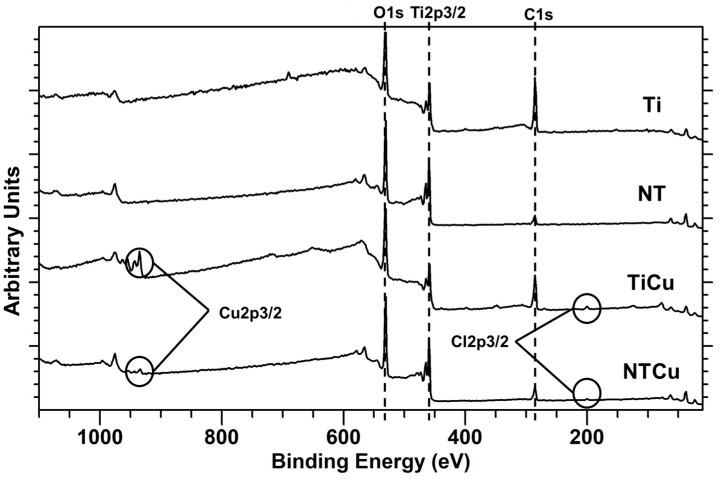
XPS survey spectra for different surfaces.

Surface chemistry plays an important role in eliciting biological responses, as well as cytotoxicity, once in contact with cells, and hence it is important to characterize it. To evaluate the surface chemistry, XPS was performed on the surfaces (Figure 3). The survey spectra were collected and further analyzed to determine the surface elemental composition using the CASA XPS software (Table 1). As expected, XPS survey spectra indicated the presence of titanium (Ti2p_3/2_), carbon (C1s), and oxygen (O1s) peaks on all the surfaces (Figure 3). The presence of carbon may be attributed to the impurities in the XPS chamber and on the surface. However, for NT and NTCu surfaces, the carbon peaks were smaller than those on Ti and TiCu surfaces (Figure 3). This was due to the etching process, which is the part of anodization that removed the impurities from the surfaces. As the carbon composition goes down, more titanium is exposed on the surface, and hence there was an increase in the titanium content for the NT and NTCu surfaces (Table 1). There was also an increase in oxygen content on the NT and NTCu surfaces. This increase was due to the oxidation of the surface during the anodization process. XPS survey spectra indicated the presence of copper (Cu2p_3/2_) peaks on TiCu and NTCu surfaces (Figure 3). On the copper-modified surfaces, a small presence of chlorine was also observed, which could have been due to the CuCl_2_ being used to deposit copper. The higher amount of copper on TiCu surfaces compared to NTCu surfaces may be due to the difference in the surface nanotopography. 

**Figure 4:**
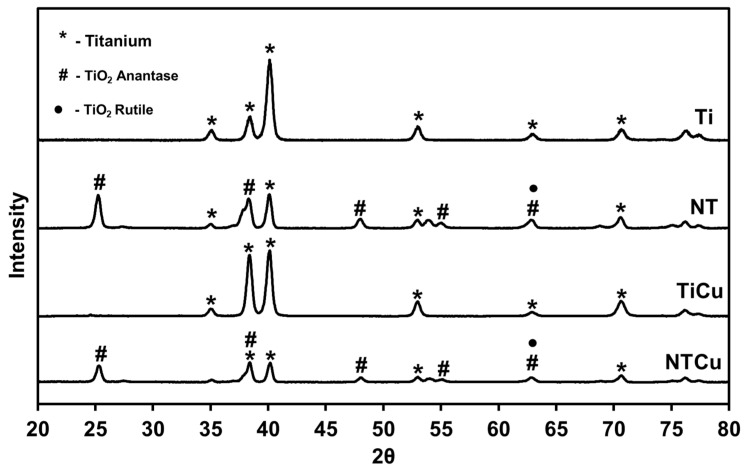
XRD spectra for different surfaces.

The surface crystallinity has a large influence on surface properties like hardness, density, and diffusion, which then eventually affects the response of cells and proteins to biomaterial surfaces [38]. To evaluate the surface crystallinity and whether the modification techniques led to changes in the crystal lattice, XRD was performed on the surfaces (Figure 4). XRD is a non-destructive analytical technique which is used to analyze crystal structure. As expected, the XRD scans indicated the presence of titanium (Ti) peaks on all the surfaces. XRD scans of both Ti and TiCu surfaces were similar, indicating that Cu modification did not alter the surface crystallinity (Figure 4). After anodization, XRD scans of NT surfaces indicated the presence of titanium oxide rutile (TiO_2_ rutile) and titanium oxide anatase (TiO_2_ anatase) phases. XRD scans of both NT and NTCu surfaces were similar, indicating that Cu modification did not alter the surface crystallinity (Figure 4). The presence of the TiO_2_ anatase phase is due to the annealing process that NT and NTCu surfaces undergo after anodization. All the surfaces (Ti and TiCu/NT and NTCu) have similar phases with varying peak intensity, which may be due to the differences in oxidation on different surfaces.

**Figure 5:**
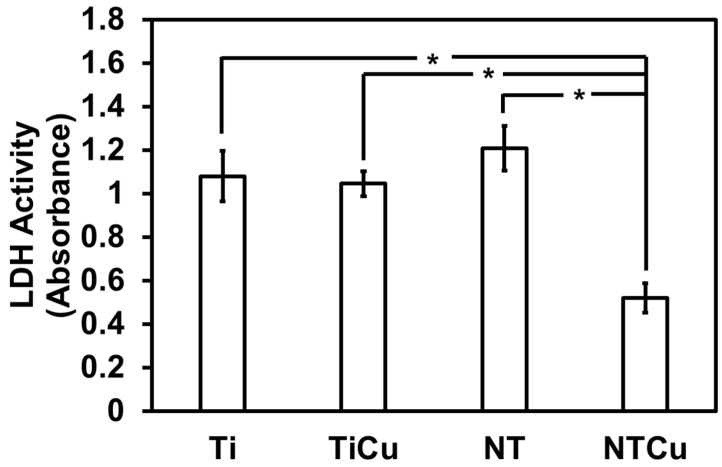
Cytotoxicity of different surfaces using LDH assay. Statistical significance (*p*-value) was represented as * *p* < 0.05.

The cytotoxicity of any material is a fundamental factor for it to be selected for implants. Hence, the cytotoxicity of different surfaces was estimated using a commercially available LDH assay. LDH is an enzyme found in cells which is released when the cells experience damage to their plasma membrane or have been lysed [39]. Higher LDH activity indicates a higher cell membrane damage and therefore that the surface is more toxic. The results showed that NTCu surfaces had a significantly lower LDH activity compared to all the other surfaces (*p* < 0.005) (Figure 5). Ti is known not to be cytotoxic to cells. There is no significant difference between Ti and NT surfaces, indicating that nanotube modification does not alter the cytotoxicity of the surfaces, and there is no significant difference between Ti and TiCU surfaces, indicating that copper modification does not negatively affect the cytotoxicity of the surfaces. 

The antifouling behavior on different surfaces was evaluated using *Staphylococcus aureus* and *Pseudomonas aeruginosa*. *Pseudomonas aeruginosa* is an opportunistic pathogen which is responsible for about 8% of the implant infections, 18.1% of pneumonias, and 16.3% of urinary tract infections, and is a major cause of cystic fibrosis [40,41]. Additionally, it is also shown to form robust biofilms and has an inherent ability to resist antibiotics [41]. 

**Figure 6:**
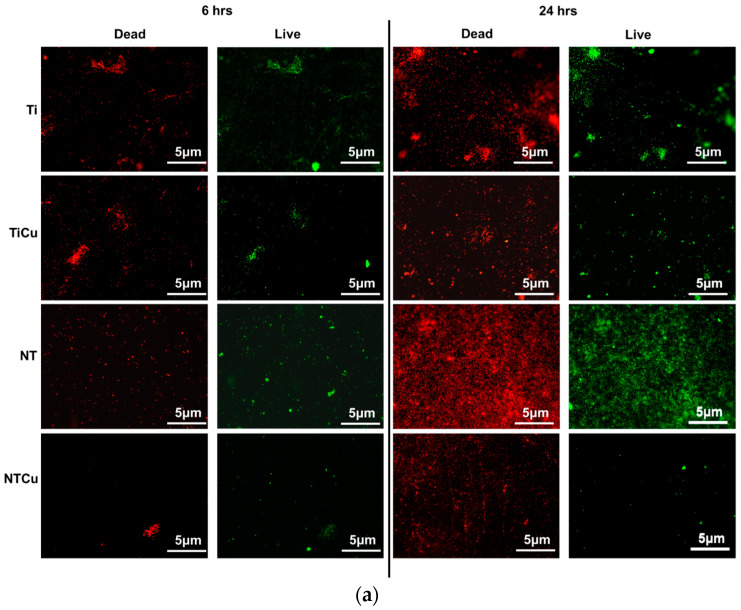

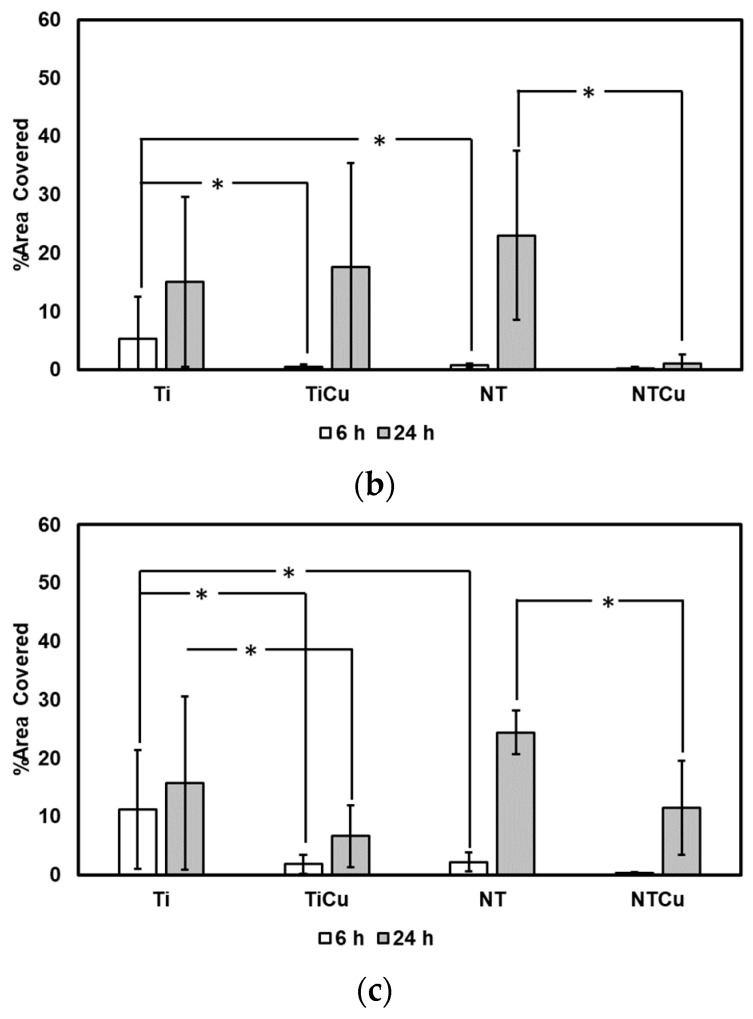
(**a**) Representative fluorescence microscopy images for *Staphylococcus aureus* adhesion and growth on different surfaces. Quantification of fluorescence microscopy images using ImageJ software indicating (**b**) live bacteria adhesion and (**c**) dead bacteria adhesion on different surfaces. Statistical significance (*p*-value) was represented as * *p* < 0.05.

To evaluate the bacteria adhesion and growth on different surfaces, fluorescence microscopy was used. Two different stains, syto9 and propidium iodide, were used to stain the adhered bacteria on different surfaces. Syto9 penetrates the cell wall, thus staining the live cells green, whereas propidium iodide is unable to penetrate cell walls and attaches itself to dead bacteria cells, thus staining the dead cells red [42]. The results for *Staphylococcus aureus* indicated increased adhesion on all the surfaces after 6 h and 24 h of culture (Figure 6a). The surfaces with copper modification, i.e., TiCu and NTCu surfaces, had lower live bacteria adhesion compared to Ti and NT, respectively (Figure 6b,c). Furthermore, Ti surfaces had the highest adhesion for live bacteria and subsequently dead bacteria after 6 h of culture. As expected, NT surfaces displayed higher dead bacteria adhesion compared to Ti surfaces, indicating the effect of topography on bacteria viability [43,44]. NTCu surfaces had the lowest adhesion of live bacteria compared to all the other surfaces after 6 and 24 h of culture (*p* < 0.05), whereas TiCu surfaces displayed the lowest adhesion for dead bacteria after 6 and 24 h of culture (*p* < 0.05) (Figure 6b,c). However, after 24 h of culture, NT surfaces displayed the highest adhesion for the live as well as dead bacteria, which has been shown by other studies as well [44]. TiCu and NTCu surfaces displayed lower adhesion for live as well as dead bacteria. Hence, these surfaces were significantly better in terms of their antifouling behavior, since they discouraged the adhesion of bacteria and killed the ones that had managed to somehow adhere on the surface. Bacterial death on these surfaces may likely occur due to the contact killing of bacteria caused due to the presence of copper. Contact killing occurs due to the damage of outer and/or inner bacterial membrane, which leads to the accumulation of copper ions in the bacteria cell, which subsequently degrades the DNA [45].

**Figure 7:**
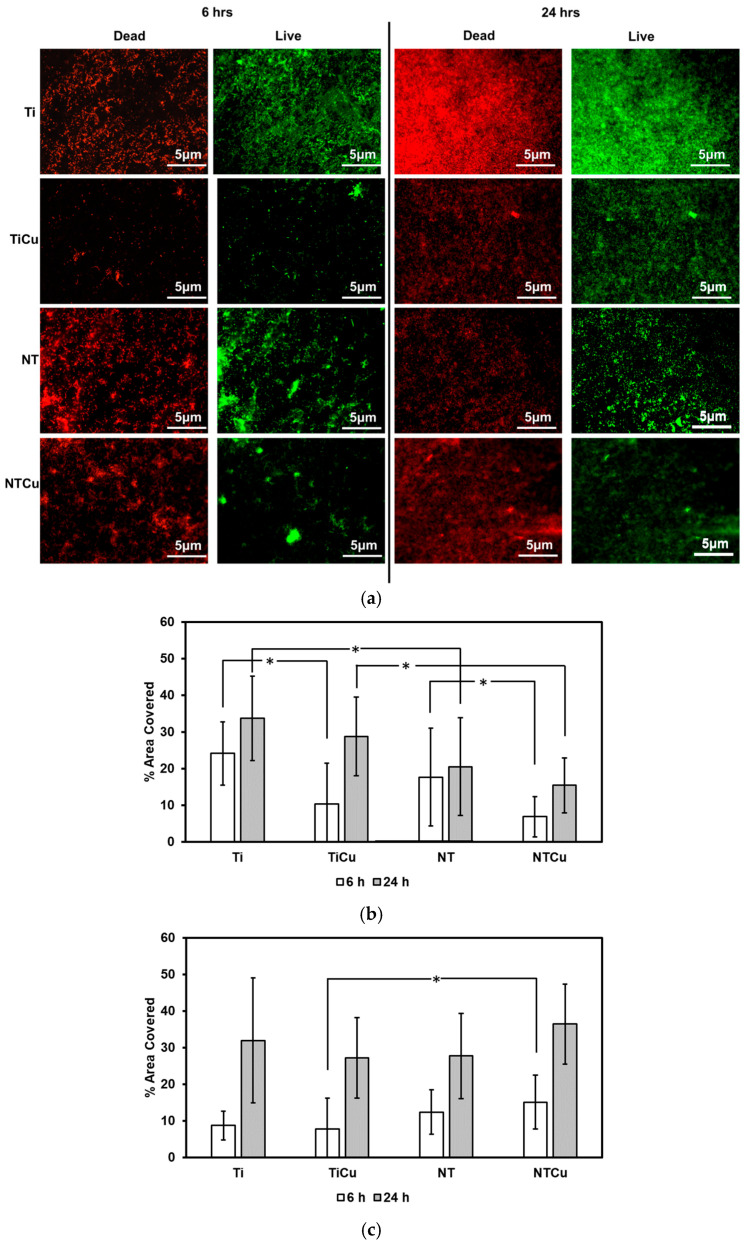
(**a**) Representative fluorescence microscopy images for *Pseudomonas aeruginosa* adhesion and growth on different surfaces. Quantification of fluorescence microscopy images using ImageJ software indicating (**b**) live bacteria adhesion and (**c**) dead bacteria adhesion on different surfaces. Statistical significance (*p*-value) was represented as * *p* < 0.05.

The results for *Pseudomonas aeruginosa* were similar to those of *Staphylococcus aureus*. Ti surfaces displayed increased adhesion for live and dead bacteria after 6 h as well as 24 h of culture (Figure 7b,c). TiCu and NTCu surfaces displayed significantly lower live bacteria adhesion as compared to Ti and NT surfaces, respectively, after 6 h as well as 24 h of culture (*p* < 0.05) (Figure 7b,c). After 24 h of culture, NTCu surfaces displayed the lowest live bacteria adhesion amongst all the surfaces and displayed the highest dead bacteria adhesion (Figure 7b,c). As described previously, the increased antifouling behavior displayed by NTCu and TiCu surfaces was likely due to the contact killing caused due to the presence of copper on the surfaces.

**Figure 8:**
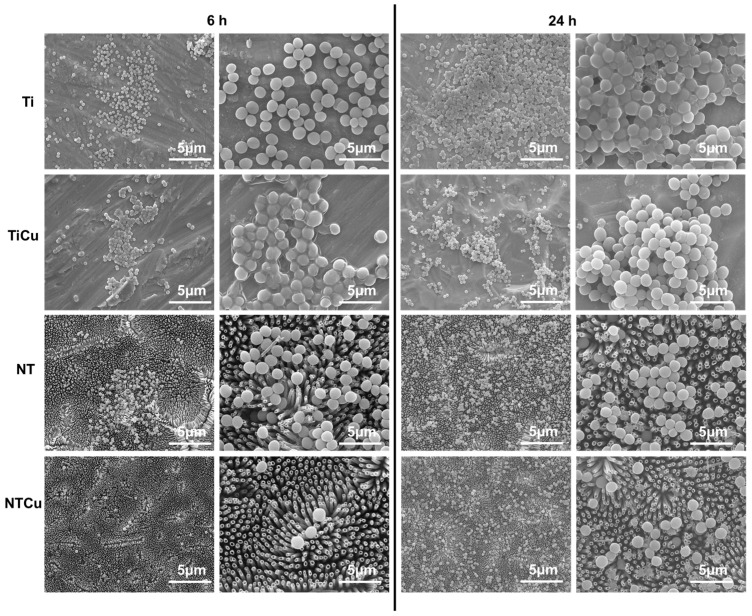
Representative SEM images for *Staphylococcus aureus* on different surfaces.

Biofilm formation is a process of irreversible attachment, colony formation, and subsequent production of extracellular polymers by bacteria. It is a grave concern for implants and is major cause for many bloodstream infections and urinary tract infections, and also enables the bacteria to develop resistance to antibiotics [27]. Hence, preventing biofilm formation is an important challenge SEM was used to evaluate the morphology and biofilm formation of bacteria on different surfaces. As expected, the SEM results for *Staphylococcus aureus* (Figure 8) were in agreement with fluorescence microscopy results (Figure 6a), indicating an increase in the number of bacteria from 6 h to 24 h of culture [42]. Ti and TiCu surfaces had a higher adhesion as well as spread of bacteria colonies across the surface, compared to NT and NTCu surfaces where there were small clusters of bacteria. Furthermore, there was some biofilm formation observed on the Ti surface for 24 h and TiCu for 6 h. NTCu surfaces displayed the lowest adhesion and growth after 6 h and 24 h of culture, and the bacteria were scattered across the surface with minimal colony formation.

**Figure 9:**
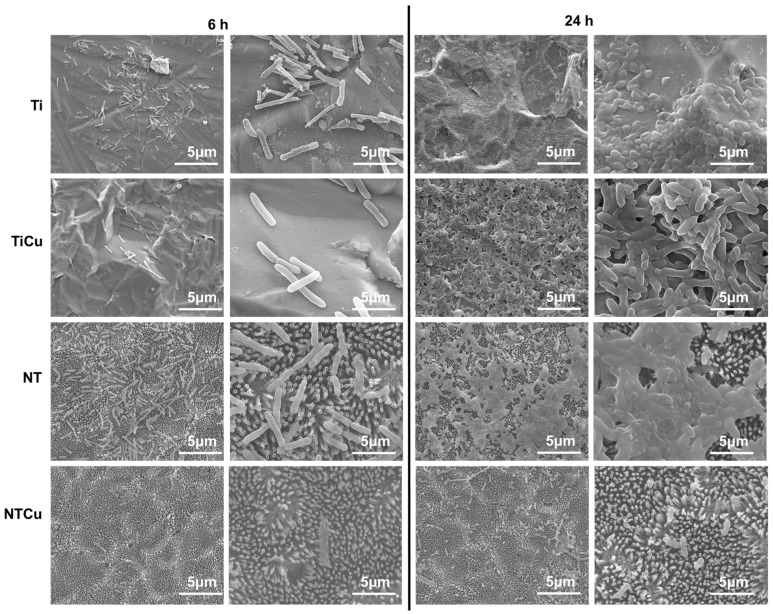
Representative SEM images for *Pseudomonas aeruginosa* on different surfaces.

The results for *Pseudomonas aeruginosa* (Figure 9) were also in agreement with fluorescence microscopy (Figure 7a) and displayed an increase in the number of bacteria from 6 h to 24 h. Biofilm formation was observed after 24 h on Ti, TiCu, and NT surfaces. The NTCu surfaces were the only ones that did not have any biofilm formation and the bacteria adhesion was very sparse on the surface. Ti and NT surfaces were completely covered with biofilm. The copper modification resulted in drastic reduction in bacteria adhesion on surfaces; however, TiCu surfaces still had some biofilm formation after 24 h. NTCu surfaces had no biofilm formation and negligible bacteria adhesion. These results indicate that copper-modified nanotube surfaces did not allow adhesion, growth, or biofilm formation for either *Staphylococcus aureus* or *Pseudomonas aeruginosa*, and thus may be a potential surface for implants.

## 4. Conclusions

Titania nanotube surfaces demonstrated increased osteoblast adhesion and a moderate decrease in bacteria adhesion. In this work, these titania nanotube surfaces were further modified with copper due to the ability of copper to contact kill bacteria. The results from SEM indicate no significant difference between the Ti/NT and TiCu/NTCu surfaces, indicating that copper modification did not alter the surface topography. Furthermore, the EDS results indicate that copper was evenly distributed on the modified surfaces. The XPS results also confirmed the presence of copper on modified surfaces. The copper-modified surfaces were also hydrophilic, indicating that they had appropriate surface proprieties for favorable protein and cell interactions. The copper-modified surfaces had a similar crystal structure to that of the unmodified surfaces. The antifouling behavior of the surfaces was evaluated using *Staphylococcus aureus* and *Pseudomonas aeruginosa* after 6 h and 24 h of culture. The results indicate the significant reduction in bacterial adhesion and absence of biofilm formation on copper-modified titania nanotube surfaces, indicating that they may be useful for orthopedic applications in preventing infection.

## Figures and Tables

**Table 1:** Surface elemental composition of the different surfaces.

	% C1s	% O1s	% Ti2p	% Cu2p	% Cl2p
**Ti**	69.13	26.42	4.39	-	-
**TiCu**	44.83	38.49	10.81	4.25	1.62
**NT**	17.50	51.78	30.72	-	-
**NTCu**	22.59	48.37	27.22	0.77	1.05
